# Scale and Ambition in the Engagement of Private Providers for Tuberculosis Care and Prevention

**DOI:** 10.9745/GHSP-D-19-00074

**Published:** 2019-03-22

**Authors:** William A. Wells

**Affiliations:** a United States Agency for International Development, Washington, DC, USA.

## Abstract

The tuberculosis (TB) community knows the importance of engaging private providers to reach critical TB targets, and knows how to engage successfully. The next challenge is to transition such efforts to government stewardship and financing in order to reach scale.

See related article by Ananthakrishnan.

## PUBLIC PROGRAMS FOR PRIVATE PROVISION

Tuberculosis (TB) programs find themselves in an unusual position. They are the quintessential public health program, focused on an airborne public health threat with many externalities beyond an individual's health, thus justifying a substantial public investment. Yet globally, one of the largest pieces of unfinished business for these public programs is to engage with private health care providers.

The reason is simple: TB is now largely concentrated in countries with large numbers of private health care providers. A recent landscape analysis of private provider engagement (PPE) in TB^1^ found that 7 of the highest TB burden countries (India, Indonesia, Philippines, Pakistan, Nigeria, Bangladesh, and Myanmar), which account for 57% of the global TB incidence and 63% of unreported (“missing”) TB cases,^2^ have dominant private sectors. In these countries, 75% (67%–84%) of initial care seeking is to private providers, and 61%–74% of total expenditure on health is private. Yet only 19% (5%–28%) of total TB notifications and 12% (1%–18%) of estimated TB incidence are notified by private for-profit TB providers.^1^

When national TB programs are not fully engaging private providers, what happens to TB patients? Indonesia and Nigeria provide contrasting experiences. In Indonesia, only an estimated one-third of the non-notified TB patients are not diagnosed; the remaining two-thirds are diagnosed but not notified.^3^ They are thus inaccessible to public interventions that would improve the quality of the 6-month treatment, reduce the likelihood of developing multidrug-resistant TB, and ensure treatment completion and success. In Nigeria, by contrast, qualitative evidence suggests that many clients of private providers do not get correctly diagnosed with TB, likely leading to extensive mortality or at a minimum delayed diagnosis, which results in ongoing transmission.

## ENGAGING COLLABORATIVELY

In this issue, Ananthakrishnan et al. report on a TB PPE project implemented in a population of 5.3 million people in Chennai, India.^4^ During 21 months of implementation, the project team engaged 227 private providers (half of those initially contacted), with an average yield of 11 symptomatics and 5 TB patients per provider, for a total of 1,232 TB diagnoses (including 26 with confirmed multidrug-resistant TB) and an overall 10% increase in TB notification in Chennai. Their approach drew from many of the lessons learned previously,^1^^,^^5^^–^^7^ such as designing the intervention in collaboration with private providers, using an “intermediary agency” to bridge between public and private, and bringing modern TB diagnostics into the private sector rather than demanding immediate referral to the public sector. The project also confirmed some previous findings, such as the occurrence of a few “super-referrers”; in their case, 9% of engaged providers supplied almost half of all referrals.

Ananthakrishnan et al. have contributed to the process of iterative learning in TB PPE, which, as the authors noted, also assisted their own work through lessons from previous donor-funded TB PPE projects in India. Their PPE effort will be expanded almost threefold by the city Corporation of Chennai. Such a process of getting past the learning stage is critical: in the past, TB PPE has been plagued by numerous small projects coming and going.^8^ Ultimately, such efforts need to go to scale—a challenge that India in particular is in the middle of confronting.

Engagement of private providers in TB has been plagued by numerous small projects coming and going.

## PAY NOW, WORRY LATER

There are two developmental strategies to this challenge of scale-up. One is to use donor financing for the implementation work now and worry about sustainability later—when the TB burden, and therefore financial need, will be lower based on the earlier, intensive effort. This is largely the approach in two substantial efforts in Pakistan and India financed by the Global Fund to Fight AIDS, Tuberculosis and Malaria (see below), though the Global Fund inputs are also subsidized by a social business model (in Pakistan^9^) and by leveraging diagnostic and drug procurements by government (in India).

Results thus far are impressive. In Pakistan, the Global Fund-financed PPE project has screened more than 3 million people for TB symptoms and detected more than 61,000 TB patients in just over 2 years.^10^ In India, the Joint Effort for Elimination of Tuberculosis (JEET) project aims to reach 49 large cities and 406 smaller cities and to engage 15,000 private doctors, resulting in an expected 1.6 million notifications over 3 years (this would include a 39% contribution to the national target of 2 million private notifications in 2020). Early progress has resulted in more than 150,000 TB notifications in the first 8 months of implementation alone (personal communication, Shibu Vijayan, PATH, 2019).

## AMBITIOUS PLANS IN INDIA

The ambition of JEET is part of an ongoing sea change in the TB response in India. The targets under India's current *National Strategic Plan for Tuberculosis Elimination, 2017–2025*^11^ start from a baseline of 11% of notifications coming from the private sector (0.19 million out of 1.74 million total notifications in 2015) and increase to a projected 56% contribution from private providers in 2020 and 2023, with 97% of the 1.86 million additional notifications in 2020 vs. 2015 projected to come from the private sector. Already, TB notifications from private providers have been steadily increasing, from 3,547 in 2012 up to 540,365 (33% of total national notifications) in 2018.^12^

It is worth quoting extensively from this *National Strategic Plan* on this change in focus^11^:

*The proposed strategy amounts to a total transformation of the way in which the programme has engaged private providers heretofore. It will be systematic and large-scale, rather than ad hoc and insignificant. It will capitalize on advances in information and communications technology and on India's drive towards digital financial inclusion. Mistrust will be replaced by constructive partnership. Rather than compete with private providers, the programme will work with them to deliver quality … services to the entire population. Rather than additionally burdening existing under-trained and over-stretched staff, the programme will contract professional agencies with the skills and capacity to engage with thousands of providers. For the first time, budgetary resources commensurate with both the problem and the opportunity of private sector care will be allocated to address the challenge.*

## TRUE SUSTAINABILITY

With the large-scale achievements of these predominantly donor-funded projects in mind, the second important strategy is to push for the sustainability of these efforts, meaning ultimately stewardship and financing by domestic governments.^13^ This includes government financing of curative TB care by private providers, often via social health insurance,^14^ with the opportunities for improved government stewardship of quality that this brings. But there also needs to be deliberate financing of the public health function as it applies to private providers. For TB, the public health function includes all of the activities—such as recording and reporting, adherence monitoring during 6 months of treatment, and contact investigation—that providers are unlikely to supply if paid primarily for curative tasks.^14^ Such functions are a particularly important input for achieving good TB outcomes. Governments can finance such a function either by hiring an expanded, dedicated PPE cadre within government itself,^15^ or by contracting out this function to the intermediaries currently supported by donor funding.^1^

Deliberate financing is needed for certain public health functions of TB care that private providers are unlikely to supply.

In the longer term, successful PPE requires the methodical development of health system steering abilities, so that governments faced with a mixed health system^16^ can govern both public and private providers equally effectively. As these competencies are being developed, a continued push for ambitious financing and achievement in the area of TB PPE remains a high priority,^17^ and is essential if the world is going to have any chance of reaching the ambitious targets^18^ arising from the United Nations High-Level Meeting on TB.

## Notes

**Disclaimer:** The views and opinions expressed in this article are those of the authors and not necessarily the views and opinions of USAID.
